# The *vapB–vapC* Operon of *Acidovorax citrulli* Functions as a *Bona-fide* Toxin–Antitoxin Module

**DOI:** 10.3389/fmicb.2015.01499

**Published:** 2016-01-06

**Authors:** Reut Shavit, Mario Lebendiker, Zohar Pasternak, Saul Burdman, Yael Helman

**Affiliations:** ^1^Department of Plant Pathology and Microbiology, The Robert H. Smith Faculty of Agriculture, Food and Environment, The Hebrew University of JerusalemRehovot, Israel; ^2^Protein Purification Facility, Wolfson Centre for Applied Structural Biology, Edmund J. Safra Campus, The Hebrew University of JerusalemJerusalem, Israel

**Keywords:** *Acidovorax citrulli*, toxin–antitoxin, VapB, VapC

## Abstract

Toxin–antitoxin systems are commonly found on plasmids and chromosomes of bacteria and archaea. These systems appear as biscystronic genes encoding a stable toxin and a labile antitoxin, which protects the cells from the toxin’s activity. Under specific, mostly stressful conditions, the unstable antitoxin is degraded, the toxin becomes active and growth is arrested. Using genome analysis we identified a putative toxin–antitoxin encoding system in the genome of the plant pathogen *Acidovorax citrulli*. The system is homologous to *vapB*–*vapC* systems from other bacterial species. PCR and phylogenetic analyses suggested that this locus is unique to group II strains of *A. citrulli.* Using biochemical and molecular analyses we show that *A. citrulli* VapBC module is a *bona-fide* toxin–antitoxin module in which VapC is a toxin with ribonuclease activity that can be counteracted by its cognate VapB antitoxin. We further show that transcription of the *A. citrulli vapBC* locus is induced by amino acid starvation, chloramphenicol and during plant infection. Due to the possible role of TA systems in both virulence and dormancy of human pathogenic bacteria, studies of these systems are gaining a lot of attention. Conversely, studies characterizing toxin–antitoxin systems in plant pathogenic bacteria are lacking. The study presented here validates the activity of VapB and VapC proteins in *A. citrulli* and suggests their involvement in stress response and host–pathogen interactions.

## Introduction

The Gram-negative bacterium *Acidovorax citrulli* is a seed-borne pathogen responsible for bacterial fruit blotch (BFB), a threatening disease of cucurbits worldwide (Schaad et al., 2003). Under favorable conditions, this bacterium spreads rapidly throughout nurseries and in the field leading to seedling blight or, at a later stage, fruit rot. Strategies for managing BFB are limited, and although seed treatments reduce disease transmission, they often fail to eradicate the pathogen from the seed (Dutta et al., 2008). In addition, chemical control of the disease in the field has only limited efficiency and to date, there are no sources of BFB resistance (Bahar et al., 2009b; Burdman and Walcott, 2012). Understanding the mechanisms that promote plant tissue colonization, virulence and spread of *A. citrulli* is therefore important for developing efficient tools to manage BFB. Based on several studies examining genetic and biochemical traits as well as host association, *A. citrulli* strains have been divided into two major groups: group I strains have been mainly isolated from various non-watermelon hosts (mainly melon), while group II strains have been generally isolated from watermelon hosts (O’Brien and Martin, 1999; Walcott et al., 2000, 2004; Burdman et al., 2005).

Using genome analysis we have identified a putative VapBC-like toxin–antitoxin (TA) encoding system in the genome of AAC00-1, a group II strain of *A. citrulli* (sequenced by the Joint Genome Institute; GenBank accession NC_008752.1). Genes encoding VapBC-like TA systems are widespread in the in the genomes of both archaea and bacteria. These systems generally appear as biscystronic genes, which encode a stable toxin (VapC), and a labile antitoxin (VapB). VapB are DNA binding proteins that can also bind the VapC toxin and inhibit its toxic activity (Robson et al., 2009). The VapC toxins are ribonucleases that belong to the PIN-domain family (a domain homologous to the N-terminal domain of the protein PilT), which usually cleave single-stranded RNA (Arcus et al., 2009; Robson et al., 2009; Winther and Gerdes, 2011). Under specific, mostly stressful conditions, the unstable antitoxin is degraded and the toxin is released from the complex leading to permanent or reversible cell growth arrest (reviewed in Hayes, 2003; Gerdes et al., 2005).

Toxin–antitoxin encoding genes are commonly found on plasmids and chromosomes of prokaryotes. While the role of plasmid-encoded TA systems as addictive modules has been extensively studied (Gerdes et al., 1986; Yarmolinsky, 1995; Engelberg-Kulka and Glaser, 1999; Cooper and Heinemann, 2000; Patel and Weaver, 2006), the physiological importance of chromosomally encoded TA systems is still under debate. A possible involvement in the following mechanisms has been proposed: (i) growth modulation under stress (Gerdes, 2000; Christensen et al., 2003; Gerdes et al., 2005); (ii) generation of persister cells (Maisonneuve et al., 2011, 2013, Gerdes and Maisonneuve, 2012); (iii) genome maintenance (Szekeres et al., 2007); and (iv) programmed cell death (Engelberg-Kulka and Glaser, 1999; Hazan et al., 2004; Mutschler et al., 2011; Erental et al., 2012). An additional hypothesized role, which relates to TA modules found in pathogenic bacteria, suggests that these systems may be involved in growth regulation of bacteria once inside the host cells (Hopper et al., 2000; Ren et al., 2012; De la Cruz et al., 2013). Due to this possible involvement in virulence, as well as in dormancy of human pathogenic bacteria, studies of TA systems are gaining a lot of attention. Recent studies present preliminary evidence suggesting that synthetic peptides can be used to modulate TA systems, thus providing avenues for the development of novel antibacterial agents (Williams et al., 2011; Williams and Hergenrother, 2012).

In contrast to the vast number of studies examining the physiological role of TA systems in animal pathogenic bacteria, almost nothing is known about the role of these systems in plant pathogenic bacteria. We report here the molecular and biochemical characterization of a VapBC-like module from the phytopathogenic bacterium *A. citrulli*.

## Materials and Methods

### Bacterial Strains, Plasmids, and Growth Conditions

Since *A. citrulli* is a quarantine bacterium in Israel, we cannot work with strain AAC00-1 that was isolated in the USA. Therefore, the study was performed using *A. citrulli* strain 7a1, a group II strain isolated in Israel (Eckshtain-Levi et al., 2014), in which the *vapBC* operon is 100% identical to that of strain AAC00-1 (see Results). Unless stated otherwise, *A. citrulli* 7a1 (Eckshtain-Levi et al., 2014) was grown in nutrient broth (NB; Difco, Franklin Lakes, NJ, USA) under constant shaking (150 rpm) or nutrient agar (NA; NB containing 15 g/l agar) at 28°C. *Escherichia coli* strains DH5α (Hanahan, 1983), BL21(DE3) and BL21-AI (Thermo Fisher Scientific^TM^, Waltham, MA, USA) were routinely grown in Lysogeny broth (LB; Difco) under constant shaking (150 rpm) or LB agar (LB containing 15 g/l agar) at 37°C. All strains were maintained as glycerol stocks at -80°C.

### General Molecular Techniques

Kits used for plasmid and PCR product extraction and purification were *AccuPrep^^®^^* Plasmid Mini Extraction Kit and AccuPrep^®^ PCR Purification Kit, respectively (Bioneer Corporation, Daejeon, Republic of Korea). DNA was extracted using the GeneElute^TM^ bacterial genomic DNA Kit (Sigma-Aldrich, St. Louis, MO, USA). RNA extraction was carried out using TRI Reagent (Sigma-Aldrich). All kits were used according to the manufacturer’s protocols unless stated otherwise. PCR products were sequenced at Hy Labs (Rehovot, Israel) and data was analyzed using the Bioedit sequence alignment editor (Tom Hall Ibis Biosciences, Carlsbad, CA, USA). Oligonucleotides primers used in this study were purchased from Sigma-Aldrich and are listed in Supplementary Table S1.

### Cloning and Sequencing of *vapBC* Genes from *A. citrulli* 7a1

Primers for amplification of vapB and vapC genes from A. citrulli 7a1 were designed according to the genome sequence of A. citrulli AAC00-1 (GenBank NC_008752) using Oligo Analyzer 3.1 (Integrated DNA Technologies Inc, Coralville, IA, USA). PCR was performed using the REDTaq ready mix (Sigma-Aldrich) in 25-μl reaction volumes. The PCR thermal profile consisted of initial denaturation for 5 min at 95°C, followed by 35 cycles each of 30 s at 95°C, annealing for 40 s at X°C, and elongation at 72°C for Y s (X and Y: annealing temperatures and elongation times, respectively, are detailed in Supplementary Table S1). A final extension step was performed at 72°C for 10 min. Samples of 5 μl from each PCR reaction were run in 1% agarose gels (w/v) for 40 min at 120 V/cm. Gels were stained with ethidium-bromide solution (0.5 μg/ml) and photographed with transmitted UV light at 295 nm. Cloning was carried out using the restriction-free cloning (RF) method as described by Unger et al. (2010). High-Fidelity DNA Polymerase Phusion^®^ (Bio Labs, New England, UK), was used in all RF PCR reactions.

### Quantitative Real-Time PCR Analyses of *vapBC* mRNA Expression Levels

Quantitative real-time PCR analyses (qRT-PCR) were performed using the StepOnePlus^TM^ Real-Time PCR System (Applied Biosystems, Foster City, CA, USA) with green Fast SYBR 2X (Applied Biosystems). Primers were designed using Primer3Input Software (v0.4.0). All values reported are given as relative expression of each gene compared to *GAPDH* mRNA expression levels. To choose the appropriate reference gene the expression levels of *GAPDH*, *16SrRNA*, and *recA* mRNA were followed under the conditions used in the qRT-PCR experiments. All samples were adjusted to 1 μg of total RNA for cDNA synthesis. mRNA levels of *16SrRNA* and *recA* changed between control and stress experiments, as indicated by changes in Q-PCR cycles, by an average of 4 and 5 cycles, respectively, whereas those of *GAPDH* did not change over than two cycles. We therefore chose to use *GAPDH* as the reference gene.

For measurements of *vapB* and *vapC* mRNA levels under antibiotic stress or starvation *A. citrulli* 7a1 cells were grown over night in LB media at 28°C, under constant shaking (150 rpm). After overnight growth, cultures were diluted 1:100 and grown up to an OD_600_ of 0.5. At this time point, 12.5 ng/μl chloramphenicol or 1 mg/ml serine hydroxamate (SHX) were added to the cultures according to the desired treatment. At various times after stress induction (0, 0.5, and 1 h), 2 ml of treated culture were centrifuged (13,000 *g*), frozen in liquid nitrogen and kept at -20°C till RNA extraction. Genomic DNA was eliminated by DNA-free DNase (Ambion, Austin, TX, USA). cDNA was generated using random primers with the High Capacity cDNA Reverse Transcription Kit (Applied Biosystems), according to the manufacturer’s instructions. Each sample contained 1 μg RNA in 20 μl of reaction mix.

For *in planta* analyses of *vapB* and *vapC* mRNA expression levels, *A. citrulli* 7a1 was grown on NA for 48 h, resuspended from plates in sterile distilled water (SDW) and adjusted to an OD_600_ of 0.5 [about 10^8^ colony forming units (CFUs)/ml] using a Helios Gamma spectrophotometer (Thermo Electron, Corp., Rochester, NY, USA). Stem inoculation experiments were performed on 8-days-old watermelon (*Citrullus lanatus*) cv. Malali (Hazera Genetics, Co., Israel) seedlings as described (Bahar et al., 2009a). Briefly, seedlings were inoculated by placing a 5-μl drop of 10^8^ CFU/ml suspensions on the hypocotyls (at approximately 1 cm above the soil). Then, a 25 gage needle was used to stab the stem through the drop. Seedlings were kept in the greenhouse (26–28°C) for 6 days. At the desired time points (6 h and 1–6 days after inoculation) 1-cm segments around the inoculation point were cut and used for RNA extraction and bacterial cell counts from inoculated plant tissue. Three pooled hypocotyl segments were used for each biological repeat. The segments were homogenized and weighted. Samples for RNA extraction were submitted to three cycles of freeze (liquid nitrogen) and thaw to ensure cell breakage. RNA extraction and cDNA preparation was carried as described above.

### Cell Growth Assays for *In vivo* Analyses of VapBC Activity

Cell growth experiments of *E. coli* BL21-AI cells expressing *vapBC* genes were carried out in 96-well microtiter plates (Thermo Fisher Scientific^TM^). The plasmid used for protein expression was pACYCDuet-1. Samples included pACYCDuet-1 plasmids expressing recombinant VapB or VapC separately, or VapB and VapC together. *E. coli* BL21-AI cells carrying an empty vector were grown as control. Each biological repeat consisted of three colonies pooled into 200 μL of LB media. Following thorough mixing, 20 μL of each sample were transferred into 180 μL LB in microtiter plates with a final concentration of 25 μg/ml chloramphenicol and 1% (w/v) glucose. Cell growth (OD_595_) was measured using an Infinite 200 PRO NanoQuant instrument and i-control^TM^ software (Tecan, Männedorf, Switzerland). Cells were grown at 37°C, and the optical density at 595 nm was measured every 15 min. The plates were shaken for a period of 15 s (linear shaking of 1 mm) before each measurement. When OD_595_ reached 0.2, protein expression was induced by 0.5% (w/v) arabinose and 1 mM IPTG (final concentrations).

### Expression and Purification of Recombinant VapB and VapC Proteins

VapB protein with an N-terminal hexa histidine-tag (6XHis tag) was expressed in *E. coli* BL21 (DE3) cells using the pET15b plasmid (Merck Millipore, Billerica, MA, USA). For expression of VapC, the VapC protein with an N-terminal 6XHis tag was co-expressed with VapB in *E. coli* BL21 AI (arabinose induced) cells using plasmid pACYCDuet-1. The *vapC* open reading frame (ORF) was inserted within the multiple cloning site 2 (MCS-2), whereas the *vapB* ORF was inserted (without a tag) within the MCS-1. Recombinant *E. coli* cells were grown at 28°C under constant shaking at 150 rpm in LB supplemented with 1% (w/v) glucose. Induction of expression was carried out when cells reached an OD_600_ of 0.5. *E. coli* BL21 (DE3) cells expressing VapB were induced by 1 mM IPTG and *E*. *coli* BL21 AI, co-expressing VapC-6xHis and VapB were induced by 1 mM IPTG and 0.5% (w/v) arabinose. Growth of induced cells continued for 3 h after which cells were centrifuged at 13,000 *g* and frozen in -20°C till protein extraction.

Cell lysis was performed by sonication with the following lysis buffer: 50 mM KH2PO4, 1.2 M NaCl, 100 mM KCl, 20% glycerol, 25 mM imidazole, and 1% tritonX100 (pH 7.8). The cell lysate was centrifuged at 11,000 *g* for 20 min at 4°C, and the insoluble fraction (consisting of insoluble VapB and inclusion bodies of VapC) was resuspended in 1% Triton (v/v) for 5 min in ice. The cell suspension was then centrifuged at 11,000 *g* for 10 min at 4°C, and the insoluble fraction was resuspended in 6 M urea and gently stirred for 1 h. Remaining particles were removed by centrifugation (11,000 *g* for 20 min) and the clarified supernatant was loaded onto a HisPur^TM^ Ni-NTA resin (Thermo Fisher Scientific^TM^) for separation of the VapC-6xHis from VapB, according to the manufacturer’s instructions. Eluted denatured protein was dialyzed in a mini GeBAflex-tube (Gene Bio-Application L.T.D, Yavne, Israel) with refolding buffer containing: 25 mM sodium phosphate buffer adjusted to pH 7.5, 10 mM imidazole, 10% (v/v) glycerol and 0.5 M NaCl. The dialyzed protein suspension was then concentrated to 1 mg/ml using Amicon Ultra-4 centrifugal filter unit with Ultracel-10 membrane (Merck Millipore, Billerica, MA, USA). Fractions containing the desired protein were analyzed by NuPAGE^®^ 4–12% Bis-Tris gels (Thermo Fisher Scientific^TM^), and were stained with InstantBlue Coommassie blue (Expedeon, Cambridge, UK), or were transferred to iBlot nitrocellulose membranes for western blot analyses, using the iBlot Gel transfer apparatus according to the manufacturer’s instructions (Thermo Fisher Scientific). VapC refolding was carried out using different buffers as described in Lebendiker and Danieli (2014). For verification of refolding efficiency we followed the turbidity of the protein suspension as described Lebendiker and Danieli (2014), as well as examined RNase activity (described below). For additional information on the procedure of western blot please see supplementary information of Supplementary Figure S2.

### *In vitro* Analyses of VapC RNase Activity

One microgram of total RNA from A. citrulli 7a1 was added to 1 μg of refolded VapC-6xHis recombinant protein in 20 μl of reaction buffer containing 50 mM Tris-HCl and 6 mM MgCl (pH 7). The reaction was allowed to proceed for 20 min at room temperature after which it was stopped by addition of 3 μl of 6x DNA loading dye (Thermo Fisher Scientific) and 1 μl of RNase inhibitor (Human Placenta RNase NEB-M0307; 40 units/μl; New England Biolabs). Control reactions included addition of the RNase Inhibitor to the reaction medium prior to the addition of RNA or addition of 10 mM EDTA to a reaction medium without MgCl. Additional control samples consisted of the buffers that were used in the reaction without VapC, these were: refolding buffer used in the dialysis, reaction buffer 50 mM Tris-HCl pH 7 and 6 mM MgCl. Samples from each reaction were electrophoresed on 1.2% agarose gels for 40 min at 120 V/cm. The gels were then stained with ethidium-bromide solution (0.5 μg/ml) and photographed with transmitted UV light at 295 nm.

### Phylogenetic Trees Analysis

VapB and VapC protein of strain AAC00-1 were BLASTed at the NCBI protein database. All results with a score > 105 were aligned with MUSCLE (Edgar, 2004) and used to create a phylogenetic tree with MEGA v6.06 (Tamura et al., 2013). The evolutionary history was inferred by using the Maximum Likelihood method based on the JTT matrix-based model. Branches with bootstrap value < 40 were collapsed.

### Statistical Analysis

All quantitative assays were analyzed using the Dunnett’s test using JMP software (SAS Institute, Inc., Cary, NC, USA).

## Results

### Genome Analysis of the Toxin–Antitoxin (TA) Locus

**FIGURE 1:**
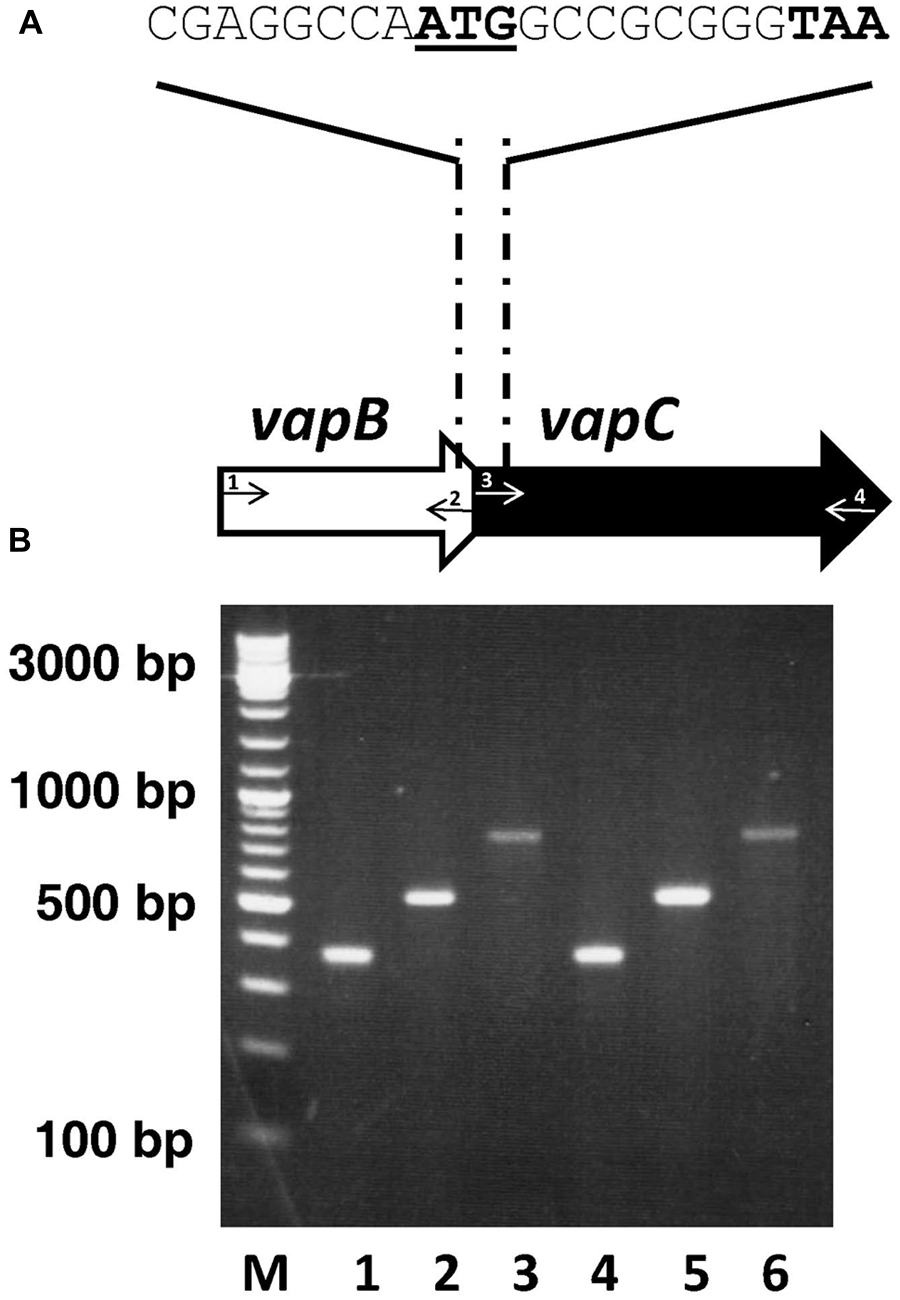
**Genetic organization of the *Acidovorax citrulli* AAC00-1 *vapBC* locus. (A)** Schematic representation of genes *vapB* and *vapC* encoding the antitoxin and toxin proteins, respectively (genes *Aave_0579* and *Aave_0580*, according to the annotation of the genome of strain AAC00-1). Sequence overlap between *vapB* and *vapC* is shown: the underlined ATG indicates the putative *vapC* start codon, while the bolded TAA indicates the putative *vapB* stop codon. Arrows and numbers indicate primer positions used for electrophoresis analysis presented in **(B)**. **(B)** Electrophoresed bands following PCR of cDNA products from *vapB* internal primers (lane 1, VapB633077 F- primer 1; VapB633412 R-primer 2); *vapC* internal primers (lane 2, VapB633292 F-primer 3; VapB633808 R-primer 4); and primers spanning the *vapB* and *vapC* ORFs (lane 3, VapB633077 F-primer 1; VapB633808 R-primer 4). Lanes 4–6, PCR-positive controls from PCR reactions using genomic DNA of strain 7a1 as template, in the same order of primers as in 1–3. Molecular weight standard of 100 base pairs increments is shown on the left (M). Negative controls with no reverse transcriptase were used to verify that RNA samples do not contain genomic DNA (data not shown). Primers are detailed in Supplementary Table S1. Results from one experiment, out of three with similar results, are shown. Expression of the *vapBC* transcript was examined after growth for 48 h in nutrient broth.

Analysis of the annotated genome of the group II strain of *A. citrulli* AAC00-1 revealed the presence of a putative *vapBC* operon in the chromosome of this bacterium (*Aave_0579* and *Aave_0580*; **Figure 1A**). Since *A. citrulli* is a quarantine bacterium in Israel, we cannot work with strain AAC00-1 that was isolated in the USA. Therefore, based on the AAC00-1 sequence we designed PCR primers that allowed us to amplify and sequence this locus from *A. citrulli* strain 7a1, a group II strain isolated in Israel (Eckshtain-Levi et al., 2014). Sequence of the TA locus of strain 7a1 revealed that it is 100% identical to that of strain AAC00-1. This sequence was deposited in the NCBI database under GenBank accessions KT149413 and KT149414 for *vapB* and *vapC*, respectively. Sequence analyses of the putative toxin gene indicated that it possibly encodes a VapC-like member of the PIN domain superfamily of ribonucleases. The putative antitoxin gene was shown to encode a transcriptional regulator/antitoxin with an AbrB-like domain commonly found in VapB antitoxin encoding genes. Sequence analyses also indicated that the putative translational start codon for *vapC* overlaps with the translational stop codon of *vapB*, providing a strong indication of translational coupling (**Figure 1A**). Reverse transcriptase (RT)-PCR experiments using primers coding for a joint segment from the end of the antitoxin and beginning of the toxin gene confirmed that these genes are expressed in *A. citrulli*, and that their expression occurs in a single transcriptional unit (**Figure 1B**).

We have recently sequenced the genome of M6, a group I strain of *A. citrulli*. Sequence analysis of the M6 draft genome revealed that it does not carry a *vapBC*-like locus. To assess whether this finding applies broadly to differences between groups I and II strains, of *A. citrulli* we employed PCR analyses to assess presence/absence of the TA locus in 15 group I strains and 12 group II strains (including M6 and 7a1, respectively). Similarly, to the results obtained from analysis of strains 7a1 and M6, the *vapBC* TA locus was shown to be present in all tested group II strains and absent in all tested group I strains (**Table 1**). Importantly, the strains selected for this analysis were isolated from various geographical locations and belong to different pulse field gel electrophoresis (PFGE)-based haplotypes (**Table 1**), thus increasing the broad significance of this finding.

**Table 1:** List of *Acidovorax citrulli* group I and group II strains examined for the presence of the *vapBC* locus by PCR analyses.

Strain name	Group	PFGE haplotype^a^	Reference/source	Toxin–antitoxin module
AACAU-2	I	B4 (L)	Walcott et al., 2004	-
AACAU-9	I	B5 (M)	Walcott et al., 2004	-
AAC98-17	I	B6 (N)	Walcott et al., 2000	-
AAC200-23	I	B8 (P)	Walcott et al., 2004	-
AAC201-16	I	B11 (V)	Walcott et al., 2004	-
AAC200-30	I	B10 (S)	Walcott et al., 2004	-
AAC92-300 (ATCC29625)	I	B3 (K)	Walcott et al., 2000	-
AAC201-15	I	B11 (V)	Walcott et al., 2004	-
AAC92-305	I	B2 (I)	Walcott et al., 2000	-
AAC201-22	I	B1 (F)	Walcott et al., 2004	-
AAC202-66	I	B12 (X)	Walcott et al., 2004	-
M1	I	B21 (Y)	Burdman et al., 2005	-
M4	I	B21 (Y)	Burdman et al., 2005	-
M6	I	B21 (Y)	Burdman et al., 2005	-
5	I	B21 (Y)	Eckshtain-Levi et al., 2014	-
AAC92-17	II	A4 (D)	Walcott et al., 2000	+
W4	II	A13 (E2)	Burdman et al., 2005	+
W6	II	A20 (Z)	Burdman et al., 2005	+
7a1	II	A23	Eckshtain-Levi et al., 2014	+
AAC94-39	II	A7 (J)	Walcott et al., 2000	+
AAC201-19	II	A2 (B)	Walcott et al., 2004	+
AAC202-69	II	A11 (W)	Walcott et al., 2004	+
AAC94-87	II	A6 (G)	Walcott et al., 2000	+
SaticoyB	II	A8 (Q)	Walcott et al., 2004	+
AAC201-20	II	A3 (C)	Walcott et al., 2004	+
AAC94-55	II	A5 (E)	Walcott et al., 2000	+
AAC94-48	II	A9 (U)	Walcott et al., 2000	+

*^a^Haplotypes based on PFGE analyses of a wide collection of *A. citrulli* strains (R. Walcott, personal communication). When available, letters inside parentheses indicate the previous haplotype designation (Walcott et al., 2000, 2004; Burdman et al., 2005).*

**FIGURE 2:**
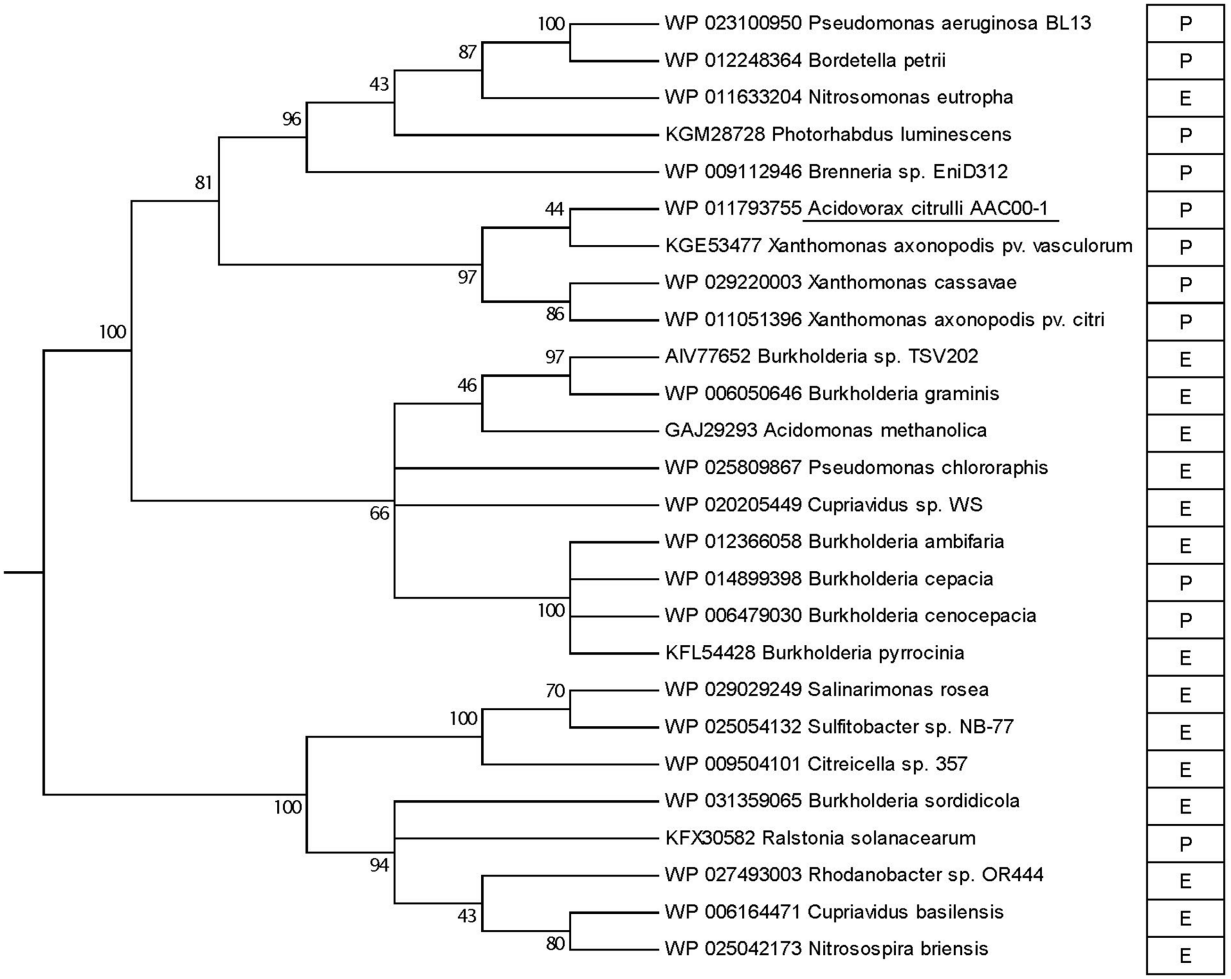
**Phylogenetic analyses of VapC-like toxin proteins.** The evolutionary history was inferred by using the Maximum Likelihood method based on the JTT matrix-based model. The percentage of trees (out of 100 bootstraps) in which the associated taxa clustered together is shown next to the branches; branches with bootstrap value <40 were collapsed. *A. citrulli* AAC00-1 is underlined. P, pathogenic; E, environmental.

Phylogenetic analysis of *A. citrulli* AAC00-1 VapC revealed that the *A. citrulli* toxin protein closely clusters with similar proteins from three *Xanthomonas* species, namely *X. axonopodis* pv. *vasculorum, X. cassavae* and *X. axonopodis* pv. *citri* (**Figure 2**). This group of *A. citrulli* and xanthomonads are branched together with a bigger cluster containing nine species, most of them pathogenic ones (eight out of nine). Phylogenic analyses of the VapB antitoxin protein of *A. citrulli* AAC00-1 revealed similar results to those of the VapC toxin, clustering it with similar proteins of other *Xanthomonas* sp. (Supplementary Figure S1). BLAST analysis of the *A. citrulli* DNA sequence containing both *vapB* and *vapC* genes, indicated that the only significant similarities found (E-value < 1) were from *Xanthomonas* strains (data not shown), which also group with the corresponding genes in the protein trees (**Figure 2**; Supplementary Figure S1).

### Ectopic Expression of the TA Module: Activity Assays and Growth Regulation

To further characterize the *A. citrulli* TA module we aimed at expressing the toxin and antitoxin proteins in *E. coli* BL21 cells. The antitoxin was successfully expressed in *E. coli* cells using the pET15b expression plasmid (Supplementary Figure S2). Repeated attempts to express the toxin on its own, using various expression plasmids in *E. coli* failed, suggesting a lethal activity of VapC (data not shown). We therefore used the pACYCDuet-1 plasmid for coupled expression of both the toxin (with His-tag) and the antitoxin (no tag). Co-expression of the antitoxin protein abolished the lethal effect of expressing the toxin alone, allowing expression of the latter in *E. coli* cells (Supplementary Figure S2).

**FIGURE 3:**
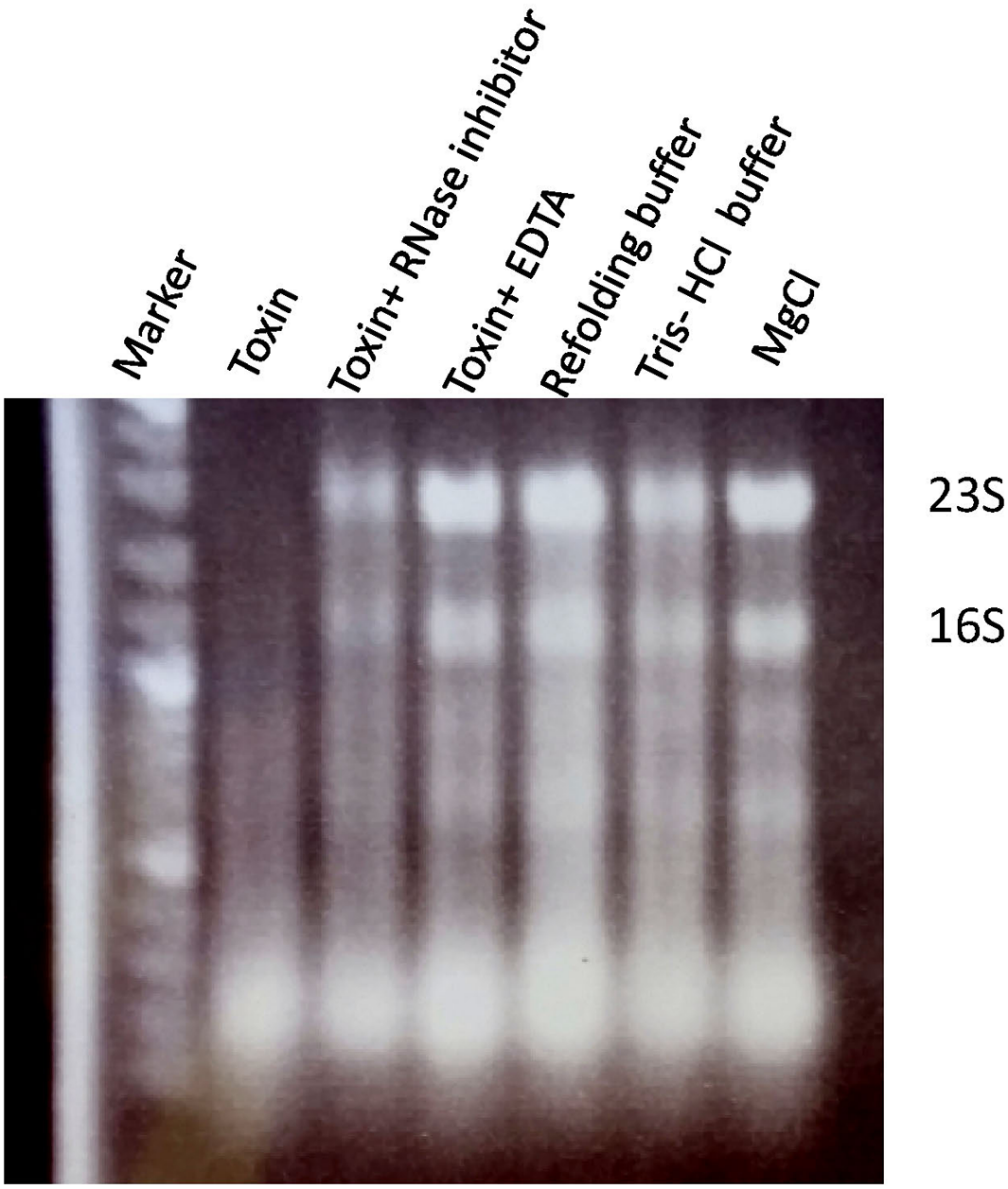
**RNase activity of recombinant *A. citrulli* VapC-6xHis protein on a preparation of cellular RNA of *A. citrulli* (1 μg/lane).** RNA was incubated for 20 min at room temperature in each treatment; Marker, (100 bp increments). Treatments: degradation of RNA by recombinant VapC-6xHis; inhibition of VapC activity by RNase inhibitor; inhibition of VapC activity by addition of 10mM EDTA; and three negative controls without VapC: refolding buffer, 50 mM Tris-HCl buffer and MgCl 6 mM solution. Results from one experiment, out of three with similar results, are shown.

The VapC PIN-domain-containing toxins are known to function as ribonucleases (Arcus et al., 2011). We therefore investigated whether the *A. citrulli* VapC protein exhibits an RNase catalytic activity. VapC from the co-purified VapB-VapC-His complex was obtained by denaturing Ni-NTA chromatography and subsequent refolding. The purified VapC-His recombinant protein efficiently degraded a cellular RNA preparation from *A. citrulli* including the 23S and 16S ribosomal RNAs (**Figure 3**). In contrast, addition of an RNase inhibitor or EDTA blocked RNA degradation (**Figure 3**), further corroborating the VapC-like nature of the toxin as a Mg^2+^/Mn^2+^-dependent ribonuclease.

**FIGURE 4:**
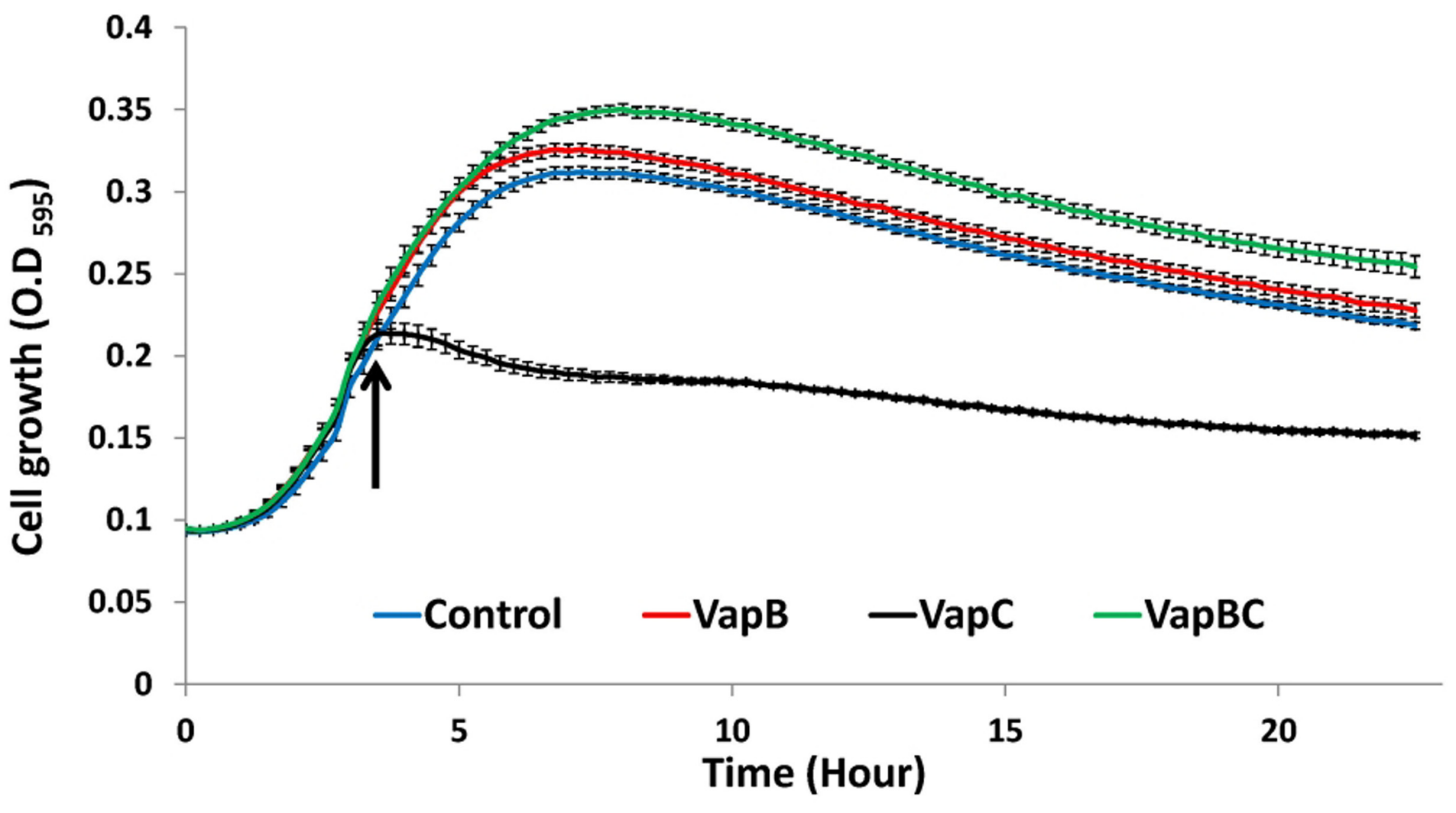
**Growth curve of *Escherichia coli* BL21-AI transformed with pACYCDuet-1 plasmids expressing recombinant VapB or VapC separately, or VapB and VapC together.** *E. coli* cells carrying the empty vector were grown as control. Cells were grown in LB media at 37°C, with linear shaking (1 mm) for 15 s every 15 min. Arrow indicates expression induction by 1 mM IPTG and 0.5% (w/v) arabinose. Growth curves were initiated by adding three colonies to each well, *n* = 12 (each replicate consisting of three pooled colonies); error bars are standard error of mean. Results from one representative experiment, out of three with similar results are shown.

Our attempts to block VapC-mediated RNA degradation by addition of the pure antitoxin VapB failed. This was probably due to the fact that during the refolding processes of the antitoxin, the protein immediately aggregated and became inactive, even when refolding was carried out together with the toxin. Nevertheless, we were able to prove that *A. citrulli* VapB and VapC form a *bona fide* toxin–antitoxin system *in vivo*: for this purpose we followed the growth of *E. coli* BL21-AI carrying a pACYCDuet-1 plasmid with (i) the antitoxin, (ii) the toxin, or (ii) both proteins together, before and after inducing expression of these genes by addition of arabinose. As seen in **Figure 4**, when expression of the toxin was induced without its cognate antitoxin, growth of the *E. coli* BL21-AI cells was arrested 15 min after arabinose induction. Conversely, and in accordance with the “antidote” activity of the VapB antitoxin, co-expression of the toxin together with its cognate antitoxin did not have any negative effect on *E. coli* growth (**Figure 4**).

### Expression of *vapBC* in *A. citrulli* 7a1

Previous studies indicated that stressful conditions such as amino acid starvation or exposure to antibiotics increase the transcription levels of the *vapBC* operon in several bacteria (Winther and Gerdes, 2009, 2012). We have conducted qRT-PCR analysis to follow the mRNA levels of the *vapBC* operon under various conditions in *A. citrulli* 7a1. Despite the fact that both genes are co-transcribed (**Figure 1B**), we measured the transcript levels of *vapB* and *vapC s*eparately. We did so since messages in polycistronic operons can often be degraded and transcribed at different rates (Arraiano et al., 2010).

**FIGURE 5:**
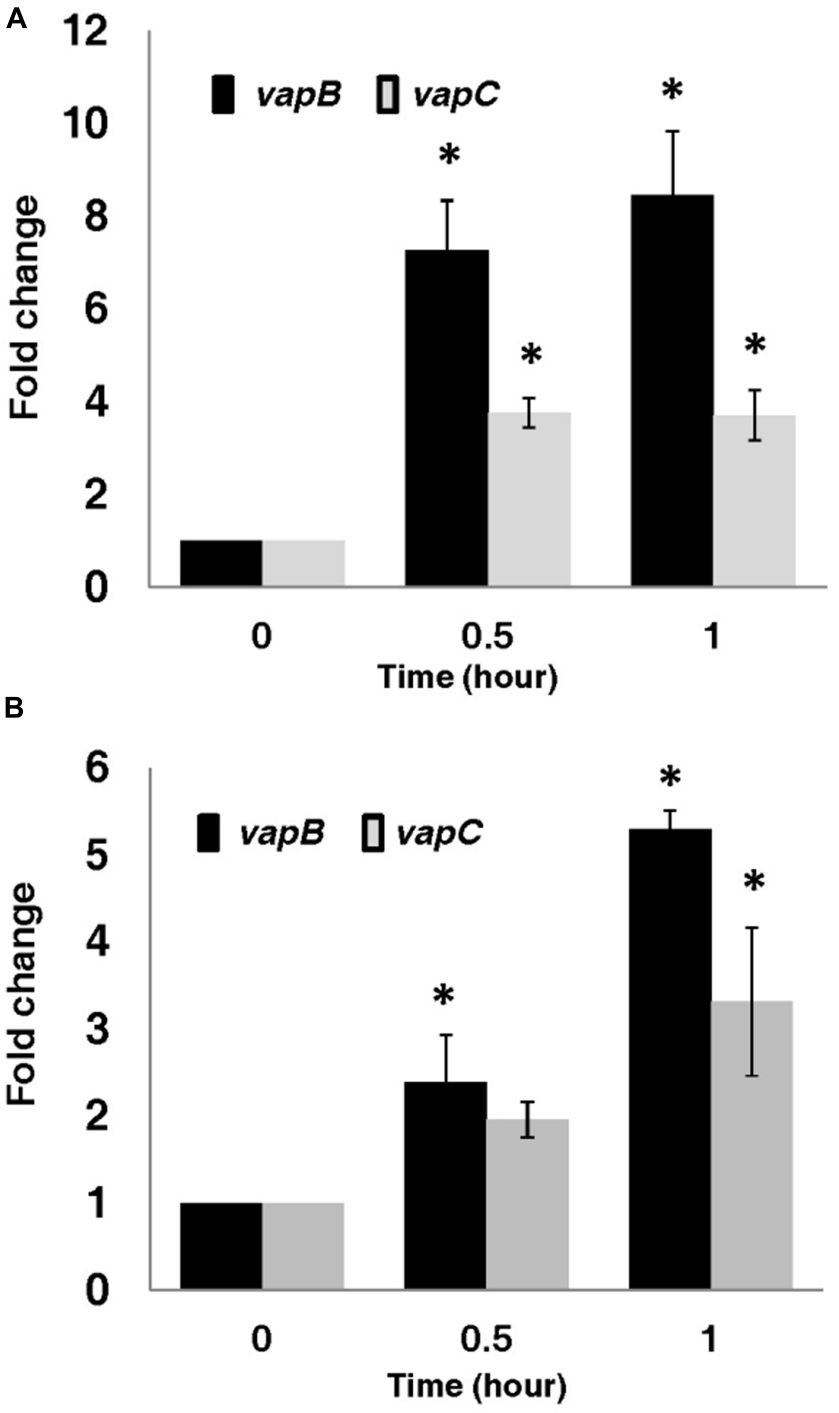
**Transcriptional activation of *vapBC* in *A. citrulli* 7a1 upon exposure to (A) antibiotic stress (12.5 μg/ml chloramphenicol) and (B) nutritional stress (1 mg/ml serine hydroxamate).** Cells were grown in LB medium containing the above stressors, with constant shaking (150 rpm) at 28°C. Black bars, *vapB*; gray bars*, vapC.* Transcription levels of these genes were measured by Quantitative-Real Time-PCR (qRT-PCR). Results represent means and standard errors of six replicates per treatment per time point. Asterisks indicate significant differences (*p* ≤ 0.05) relative to time zero for each gene, according to ANOVA and the Dunnett’s *post hoc* test. Results from one experiment, out of three with similar results, are shown.

Our results show that, in line with previously described TA systems, exposure of *A. citrulli* 7a1 to chloramphenicol (Cm; 12.5 μg/ml) resulted in a significantly (*p* ≤ 0.05) increased transcription of the *vapBC* locus (**Figure 5A**). Increased expression of the operon was detected after 30 min of growth in the presence of the antibiotic. Notably, the increase in *vapB* transcript level was higher than that of *vapC*. At 30 min of exposure to Cm, the mRNA levels of the *vapB* antitoxin were about seven times higher (7.3 ± 1) than those measured at time zero, while the mRNA levels of the *vapC* toxin increased by about four folds (3.8 ± 0.3). After 1 h of exposure to Cm, *vapB* mRNA levels increased by 8.5 ± 1.4 and those of *vapC* increased by 3.7 ± 0.5 in comparison to those measured at time zero (**Figure 5A**).

Induction of *vapBC* also occurred when *A. citrulli* cells were exposed to amino acid starvation imposed by the addition of SHX, an inhibitor of seryl-tRNA charging (Tosa and Pizer, 1971). After 30 min of exposure to SHX a significant (*p* ≤ 0.05) increase was recorded only for *vapB* (2.4 ± 0.55). Nevertheless, after 1 h of exposure to SHX both *vapB* (5.3 ± 0.22) and *vapC* (3.3 ± 0.8) transcript levels significantly (*p* ≤ 0.05) increased compared to time zero (**Figure 5B**).

**FIGURE 6:**
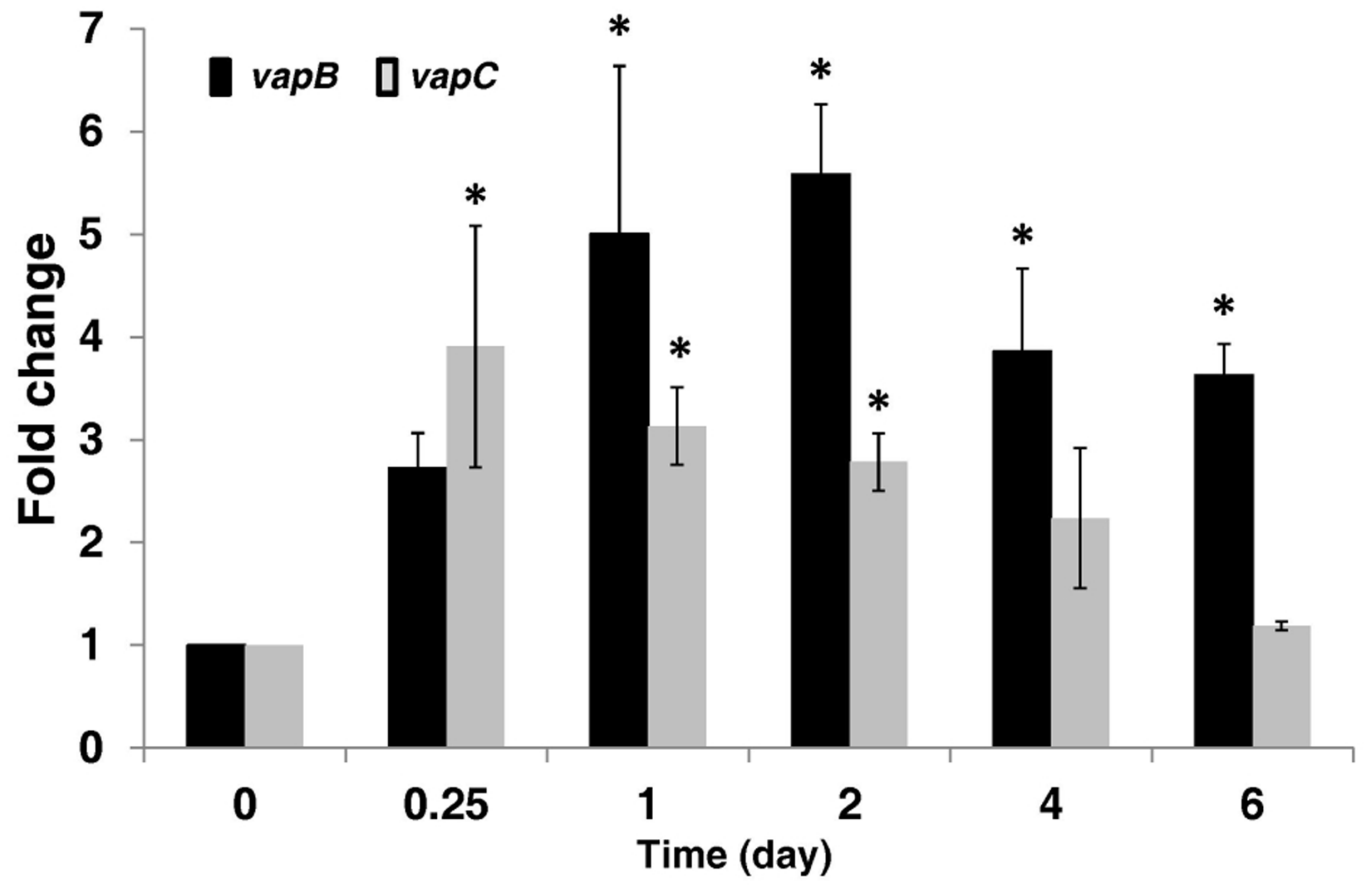
**Transcriptional activation of the *A. citrulli* 7a1 *vapBC* operon *in planta*.** Eight-days-old watermelon (cv. Malali) seedlings were inoculated with *A. citrulli* 7a1 by stem inoculation as described in Experimental Procedures. Inoculated seedlings were kept in a greenhouse at 26–28°C. At each time, seedlings were collected and RNA was extracted from 1-cm hypocotyl segments around the inoculation sites. Transcription levels of *vapB* and *vapC* were measured by qRT-PCR. Black bars, *vapB*; gray bars*, vapC.* Results represent means and standard errors of three replicates per treatment per time point, each replicate consisting of three pooled hypocotyl segments. Asterisks indicate significant differences (*p* ≤ 0.05) relative to time zero for each gene, according to ANOVA and the Dunnett’s *post hoc* test. Results from one experiment out of two with similar results, are shown.

One of the hypothesized roles for TA systems is that they are used to regulate the growth of pathogens once inside the host. We therefore followed the transcription levels of *A. citrulli vapBC* genes at various time points after stem inoculation of melon seedlings, performed as described (Bahar et al., 2009a). Our results indicate that the *A. citrulli vapBC* operon is activated during the infection process (**Figure 6**). In contrast to animal pathogens, most plant pathogenic bacteria, including *A. citrulli*, colonize extracellular spaces within the plant tissue (Alfano and Collmer, 1996). Therefore, these results correspond to interaction of the bacteria with the plant tissue and not to an intracellular behavior. At 6 h after inoculation, *vapC* levels increased significantly (*p* ≤ 0.05) as compared to *vapC* levels at time zero (3.9 ± 1.2). At this time, a higher level of expression relative to time zero was measured for *vapB* (2.7 ± 0.3), though not significant. At longer time points, the levels of *vapB* transcripts increased more than those of *vapC*. At 1 and 2 days after inoculation (d.a.i.), *vapC* levels were still significantly (*p* ≤ 0.05) higher than those at time zero but they hardly changed (3 ± 0.4 and 2.8 ± 0.3, respectively), whereas those of *vapB* continued to increase during these days (5 ± 1.6 and 5.6 ± 0.7 folds, respectively). A trend was observed indicating reduction of *vapC* expression with time, and at 4 and 6 d.a.i., the expression of this gene was not significantly different from that measured at time zero (2.2 ± 0.7 and 1.2 ± 0.04 folds, respectively). In contrast, at 4 and 6 d.a.i. *vapB* levels were still significantly (*p* ≤ 0.05) higher relative to time zero (3.9 ± 0.8 and 3.6 ± 0.3 folds, respectively). Notably, beyond 6 d.a.i, the seedlings were severely affected by the bacterium and accurate sampling was not possible beyond this time. Importantly, although the transcript levels of the *vapBC* operon increased during the first 2 days of infection, bacterial counts increased from ∼8 × 10^5^ CFU/cm hypocotyl at the inoculation time to approximately 1.3 × 10^8^ and 4.4 × 10^8^ CFU/cm hypocotyl at 2 and 4 d.a.i, respectively, thus indicating that no apparent growth arrest of *A. citrulli* occurred during the time of *vapC* expression.

## Discussion

We identified a VapBC-like toxin–antitoxin module in the genome of the group II strain of *A. citrulli*, AAC00-1. Genetic analysis of the *vapBC* locus from various *A. citrulli* strains, with distinguished genetic backgrounds, supports this locus exists only in group II strains of *A. citrulli*. Sequence analyses of the *A. citrulli vapBC* locus revealed that the only significant similarities (E-value < 1) from the available database are from several *Xanthomonas* species and pathovars and this is supported by phylogenetic relatedness at the VapB and VapC protein levels. These results suggest that the *vapBC* in group II strains of *A. citrulli* and in some *Xanthomonas* species were obtained through horizontal gene transfer. In *A. citrulli*, acquisition of this TA module possibly occurred after the splitting of this species into group I and group II strains. The opposite possibility- namely, that the *vapBC* operon was present in group I *A. citrulli*, but lost through time- cannot be discarded but is less likely due to the stabilizing addictive characteristics of TA gene systems (Hayes, 2003; Magnuson, 2007; Saavedra De Bast et al., 2008).

The high relatedness between *A. citrulli* and xanthomonads *vapBC* sequences is interesting but not surprising. For instance, based on genetic composition and regulation, *A. citrulli* and *Xanthomonas* species possess a highly similar type III secretion apparatus (Bahar and Burdman, 2010). Moreover, a recent study revealed that the majority of genes encoding putative type III secreted effectors in *A. citrulli* are highly similar of known xanthomonads effectors (Eckshtain-Levi et al., 2014). Due to the common phytopathogenic nature of *Xanthomonas* and some *Acidovorax* species, it is plausible to assume that horizontal gene transfer events have occurred among member of these genera or among ancestral species.

In this study we present molecular and biochemical evidence demonstrating that the *A. citrulli vapBC* genes indeed encode a *bona fide* VapBC TA module. We show that ectopic expression of VapC toxin in *E. coli* succeeded to inhibit cell growth and that this inhibition was counteracted by the expression of the cognate VapB antitoxin. We also showed that, as suggested by the presence of a PIN domain sequence, VapC indeed has ribonuclease activity, efficiently degrading a cellular RNA preparation from *A. citrulli*, including the 23S and 16S ribosomal RNAs. Characterization of VapC proteins from the enteric pathogenic bacteria *Salmonella enterica* and *Shigella flexneri* revealed they do not cleave mRNAs but rather act as site-specific riboendonucleases that cleave initiator tRNA fMet in the anticodon loop (Winther and Gerdes, 2009, 2011). In contrast, McKenzie et al. (2012) showed that VapC of *Mycobacterium smegmatis* cleaves RNA oligonucleotides at AUAA and AUAU sequences yielding a 5′ phosphate on the 3′ cleavage product. Additionally, VapC-1 from non-typeable *Haemophilus influenzae* was shown to cleave single-stranded RNA and the VapC-mt5 toxin protein complex from *Mycobacterium tuberculosis* appeared to cleave dsRNA (Arcus et al., 2005; Daines et al., 2007; Miallau et al., 2009). These seemingly contradictory results suggest that the mode of action of different VapC-like proteins may vary among different members of this family.

Transcription levels of TA operons are commonly used as an indirect read-out of TA system activation. This is due to the fact that in most TA systems the transcription of the TA operon is autoregulated by the antitoxin, which acts as a transcription repressor. The toxin in turn can act as a co-repressor, whereby its binding to the antitoxin strengthens the interaction between the antitoxin and DNA (Afif et al., 2001; Christensen et al., 2001; Robson et al., 2009). Under various, mostly stressful conditions, degradation of the unstable antitoxin is enhanced (commonly by lon or clp proteases), thus relieving its transcriptional inhibitory activity, as well as “freeing” the more stable toxin to interact with its cellular targets (Hazan et al., 2004; Bodogai et al., 2006; Arcus et al., 2009; Winther and Gerdes, 2009). Accordingly, we showed that transcription of the *A. citrulli vapBC* locus was induced by amino acid starvation and exposure to chloramphenicol. Similar results were reported by Christensen et al. (2001, 2003) and Winther and Gerdes (2009) who demonstrated that transcriptions of the *relBE*, *chpAK*, *chpBK, and hicAB* TA systems in *E. coli*, were induced during SHX nutritional stress and chloramphenicol exposure, as well as that of the *vapBC* operon in *S. enterica* and *S. flexneri* plasmid pMYSF6000 (Winther and Gerdes, 2009). In our experiments, the fold increments measured in transcript levels under the various stresses were significant, and in the order of ∼2 to 8 folds. Similar levels of induction were also reported for (i) *hicAB* of *E. coli* (∼12 and ∼15 fold) in response to chloramphenicol and SHX (Jørgensen et al., 2009), (ii) for the toxins *relE1-3* of *M. tuberculosis* (∼2 to 11 fold) in response to the antibiotics rifampin, gentamicin, and levofloxacin (Singh et al., 2010), and (iii) for the antitoxins *yefM and dinJ* (∼3–5 folds) in *E. coli* in response to overexpression of the global regulator Hha (García-Contreras et al., 2008).

An interesting result stemming from our qRT-PCR analyses is that there is a significant difference in transcription induction between the toxin and antitoxin under the different examined conditions. Such differences between transcription levels of *vapB* and *vapC* were also documented in transcriptome analyses of the archaeon *Sulfolobus solfataricus* (Cooper et al., 2009). The level of mRNA transcripts of a specific gene is determined by the efficiency of transcription, stability of the mRNA, and the frequency of translation. The half-life of a particular mRNA can fluctuate widely, thus changing the level of the transcript without any alteration in transcription rate. Even messages physically linked together in polycistronic operons could be degraded independently at different rates (Alifano et al., 1994; Regnier and Arraiano, 2000; Arraiano et al., 2010). In TA modules the translation rate for the antitoxin has been found to be higher than the one for the toxin (Gerdes and Maisonneuve, 2012). Such regulation is important because the antitoxins are unstable compared to their cognate toxins and therefore in order to refrain from the toxin activity, the antitoxins should be translated in a higher rate. Our results suggest that there might also be a pathway regulating the ratio toxin/antitoxin through changes in the rate of the mRNA transcription/degradation in addition to that of protein translation rate or the degradation rate of antitoxins by specific proteases.

Further experiments using antibodies against both the toxin and antitoxin, and comparison between protein and mRNA levels, are necessary to fully elucidate this possible regulation pathway.

In recent years there is increasing evidence implying that TA modules are involved in host-pathogen interactions. Recently, Georgiades and Raoult (2011) systematically compared the genomes of the 12 most dangerous pandemic bacteria for humans (“bad bugs”) to their closest non-epidemic related species (“controls”). Their results showed that the “bad bugs” have surprisingly more TA modules than do the “controls,” further supporting a role in pathogen-host interactions for the TA systems. Additionally, Bodogai et al. (2006) suggested that NtrPR, a VapBC-like module of *Sinorhizobium meliloti*, contributes to adjusting metabolic levels under beneficial symbiosis with plants. In our study, in addition to increased transcription in response to stress, we also demonstrate that the *A. citrulli vapBC* operon is induced during the plant infection process. The fact that mRNA levels of *vapB* and *vapC* are increased upon inoculation suggests a possible role for this system in host-pathogen interactions. Despite the observed activation of the *vapBC* operon *in planta* during the first 2 days after inoculation, no growth inhibition of *A. citrulli* was observed within the plant at these stages. It is possible that the observed increase in *vapBC* expression occurred only in a small proportion of the bacterial population and therefore, it is not reflected in overall growth parameters. Indeed, several studies show that heterogeneous activation of the TA system increases the percentage of persistent cells within a population, thereby providing individuals within the colony with means to avoid the damage caused by stress and host defense responses (Maisonneuve et al., 2011, 2013; Fasani and Savageau, 2013). In this regard, a study by De la Cruz et al. (2013), who examined the involvement of the TA system in the virulence of *Salmonella enterica* subsp. *enterica* serovar Typhimurium (*S.* Typhimurium) in mice, showed that increased expression of the TA module in *S.* Typhimurium is transient upon infection and that expression varies between different locations within the host. The authors also showed that the toxin itself is necessary but not sufficient to limit bacterial growth and that an additional factor synergizes with the toxin activity under conditions of mouse infection. Additionally, in a recent study presented by Lobato-Márquez et al. (2015) it was shown that both type I and type II toxins are essential for survival of *S.* Typhimurium inside fibroblasts, irrespective of the growth rate. Interestingly, the authors showed that a *vapC* mutant of *S.* Typhimurium exhibited up to 80% decrease in the rate of intracellular survival compared to the wild-type strain. Their results suggest that there is a specialization of distinct TA modules for regulating intracellular activity of pathogenic bacteria, and thus progression of infection.

Further studies examining the expression of the *vapBC* loci in different locations within the plant and in other tissues are required to further understand the role of this system in plant infection. Additionally, construction and characterization of *A. citrulli* mutants impaired in the expression of the TA module could provide insights as to the possible role of this system in such interactions. Albeit, it should be noted that in some studies of various TA systems no apparent phenotype of TA mutants was ever detected, although overexpression of the toxin component negatively affected growth *in vitro* (Tsilibaris et al., 2007; Robson et al., 2009).

Characterization of TA modules in plant pathogenic bacteria is lacking behind that of human pathogens. Here we show that *A. citrulli* possesses a TA module similar to that of known human pathogens such as *M. tuberculosis, Salmonella*, and *H. influenzae*. To the best of our knowledge, this is the first report showing expression of a TA module during infection of a plant pathogenic bacterium. We aim to further examine the mechanism, role and activation pathway of the *A. citrulli* TA module upon plant infection. Moreover, due to the presence of TA modules in other plant pathogenic species, and particularly in several xanthomonads, the relevance of the findings presented in this study is much beyond the *A. citrulli*-cucurbit pathosystem. Further studies will provide important insights into the role of TA systems in plant-microbe interactions, as well as provide valuable information on the regulation and function of TA systems in microbial populations in general.

## Conflict of Interest Statement

The authors declare that the research was conducted in the absence of any commercial or financial relationships that could be construed as a potential conflict of interest.
