# Supplementary material for: The vapB–vapC Operon of Acidovorax citrulli Functions as a Bona-fide Toxin–Antitoxin Module
Source: Front Microbiol. 2016 Jan 6;6:1499. doi: 10.3389/fmicb.2015.01499 (PMC4701950; doi:10.3389/fmicb.2015.01499)
Supplement: Supplementary file 1 [file Table_1.DOCX]

**Table S1.** Sequence of primers used in this study

| **Primer set** | **Primer sequence (5’–3’)^a^** | **Description of PCR product and size** | **Annealing (°C)** | **Elongation time (s)** |
| --- | --- | --- | --- | --- |
| VapB-RT- F  VapB- RT- R | GTAGTCGAAGCGCTCGAACTG  TTGCTCACCGCGAACGAT | 150-bp internal fragment of *vapB* for quantitative real time PCR (qRT-PCR) analysis | 60 | 30 |
| VapC- RT- F  VapC- RT -R | GCGTGCGCAAGCTGAAG  GCGGAACAATGCTGCAGAA | 150-bp internal fragment of *vapC* for qRT-PCR analysis | 60 | 30 |
| GAPDH- RT- F  GAPDH –RT- R | TCGGCCGTGCAGAACTTC  TGCAGCATGTAGGCCAGGTA | 82-bp internal fragment of GAPDH for qRT-PCR analysis | 60 | 30 |
| VapB633077 F  VapB633412 R | GCCTTTGCCATTTGTCGC  GTTGCTGTCGAGGAACACCTTG | 335- bp fragment of *vapB* used to amplify DNA and cDNA fragments | 55 | 30 |
| VapC633292 F  VapC633808 R | TTCGCGGTGAGCAAGAAGC  CATGCTGCGTCAAACGTTG | 516- bp fragment of *vapB* used to amplify DNA and cDNA fragments | 57 | 45 |
| VapB633077 F  VapC633808 R | GCCTTTGCCATTTGTCGC  CATGCTGCGTCAAACGTTG | 730- bp fragment of *vapB* and *vapC* used amplify DNA and cDNA fragments | 56 | 45 |
| VapB pET15b F  VapB pET15b R | CACAGCAGCGGCCTGGTGCCGCGCGGCAGCATGCGCTGCGGAAGGCTTAGG  GGCCCCAAGGGGTTATGCTAGTTATTGCTTACCCGCGGCCATTGGCC | 350-bp fragment of *vapB* with overhang of MCS of pET15b plasmid. For restriction free plasmid construction | 62 | 30 |
| VapC Duet F  VapC Duet R | GCAGCAGCCATCACCATCATCACCACAGCCAGGCCGCGGGTAAGGGCAAGG  CGATTACTTTCTGTTCGACTTAAGCATTATGCTCAAACGTTGAACGGGTTACG | 530-bp fragment of *vapC* with overhang of MCS-1 of pACYCDuet-1plasmid  For restriction free plasmid construction | 67 | 30 |
| VapB Duet F  VapB Duet R | GGAGATATACATATGGCAGATCTCAATTGGCGCTGCGGAAGGCTTAGGATAT  GCAGCGGTTTCTTTACCAGACTCGAGGGTACCCCCGCGGCCATTGGCCTCG | 350-bp fragment of *vapB* with overhang of MCS-2 of pACYCDuet-1plasmid  For restriction free plasmid construction | 69 | 30 |
