# Supplementary material for: The vapB–vapC Operon of Acidovorax citrulli Functions as a Bona-fide Toxin–Antitoxin Module
Source: Front Microbiol. 2016 Jan 6;6:1499. doi: 10.3389/fmicb.2015.01499 (PMC4701950; doi:10.3389/fmicb.2015.01499)
Supplement: Supplementary file 2 [file Image_1.PDF]

Supplementary information

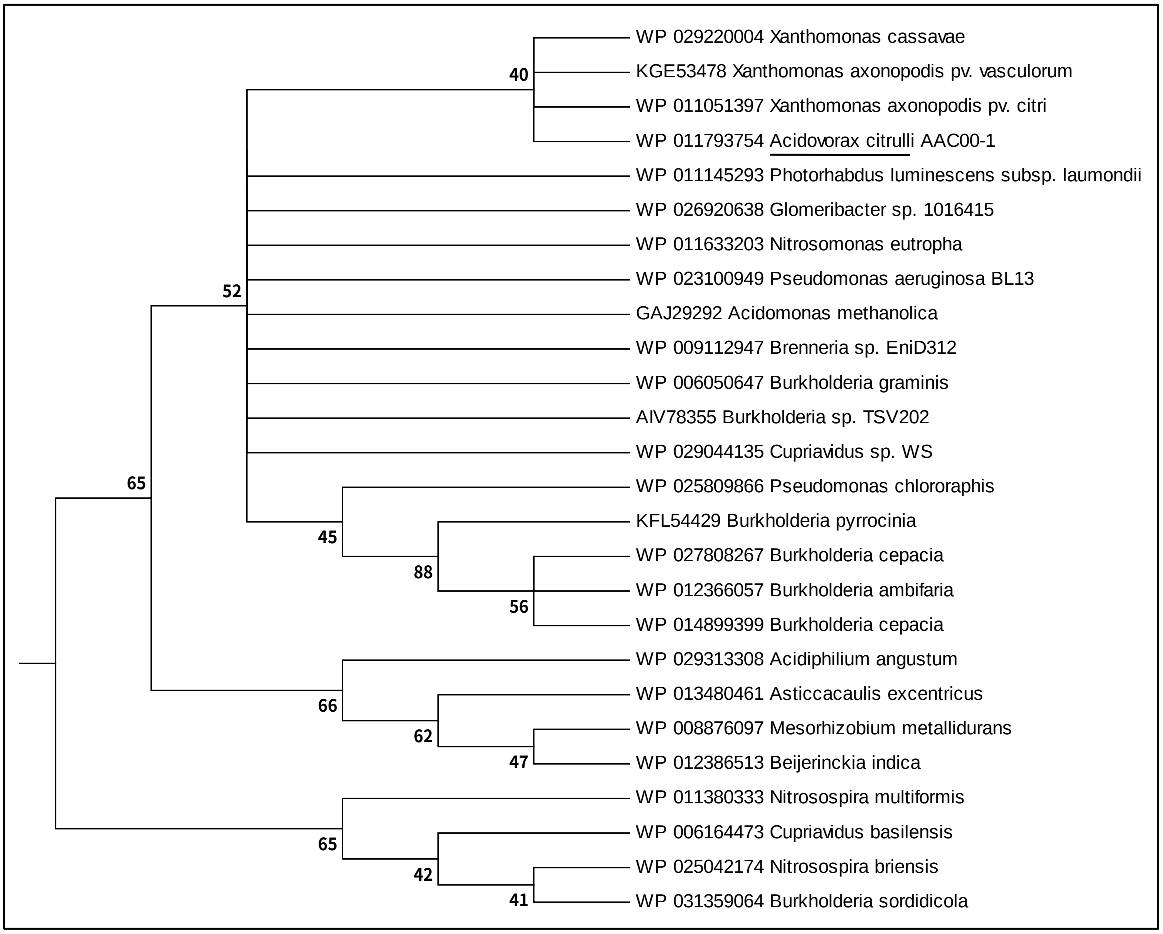

Figure S1. Phylogenetic analyses of the antitoxin VapB protein of *Acidovorax citrulli* AAC00-1. The evolutionary history was inferred by using the Maximum Likelihood method based on the JTT matrix-based model. The percentage of trees (out of 100 bootstraps) in which the associated taxa clustered together is shown next to the branches; branches with bootstrap value <40 were collapsed. *A. citrulli* AAC00-1 is underlined.
