# Supplementary material for: The vapB–vapC Operon of Acidovorax citrulli Functions as a Bona-fide Toxin–Antitoxin Module
Source: Front Microbiol. 2016 Jan 6;6:1499. doi: 10.3389/fmicb.2015.01499 (PMC4701950; doi:10.3389/fmicb.2015.01499)
Supplement: Supplementary file 3 [file Image_2.PDF]

Supplementary information

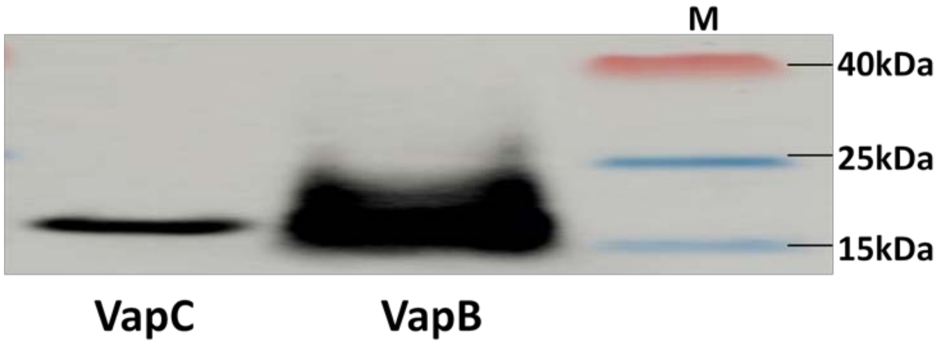

Figure S2. Western blot analyses of the His-tag recombinant proteins VapB and VapC. VapB was expressed in BL21(DE3) carrying the pET15b plasmid and VapC was expressed in *E. coli* BL21 AI carrying the pACYCDuet-1 plasmid. Proteins were blotted after elution from the HisPur™ Ni-NTA resin column. Detection was carried out with anti-His 6x monoclonal antibodies (1:1,000; BioRad) and horseradish peroxidase anti-mouse IgG (1:10,000; Santa Cruz Biotechnology), as primary and secondary antibodies, respectively. Lane M contains molecular-weight markers labeled in kDa.

Experimental procedure -western blot analyses:

Fractions containing the desired protein were analyzed by NuPAGE® 4-12% Bis-Tris gels (Thermo Fisher Scientific™) and were transferred to iBlot nitrocellulose membranes for western blot analyses, using the iBlot Gel transfer apparatus according to the manufacturer's instructions (Thermo Fisher Scientific™). Membranes were incubated for 1 h in blocking solution consisting of 3% (w/v) skimmed milk powder in T-TBS (20 mM

Tris-HCl, pH 7.5, 0.9% NaCl (w/v), and 0.1% (v/v) Tween 20). The membranes were then incubated in the presence of anti-His 6x monoclonal mouse antibodies (Bio-Rad Laboratories Inc, Hercules, CA, USA) at a dilution of 1:1,000 in blocking buffer over night at 18°C. Following three 10-min washes in T-TBS, the membranes were incubated for 1 h at room temperature in blocking buffer containing 1:10,000 dilution of goat anti-mouse antibody coupled to horseradish peroxidase (Santa Cruz Biotechnology, Dallas, Texas, U.S.A.). After three washes as described above, the membranes were incubated for 1 min with SuperSignal West Pico Chemiluminescent Substrate (Thermo Fisher Scientific™) and were developed by autoradiography using the Image Quant Las 500 (GE healthcare Bio- Sciences AB, Uppsala, Sweden).
